# Hepatitis B Virus Vaccination in HIV-1-Infected Young Adults: A Tool to Reduce the Size of HIV-1 Reservoirs?

**DOI:** 10.3389/fimmu.2017.01966

**Published:** 2018-01-10

**Authors:** Yonas Bekele, Rebecka Lantto Graham, Sandra Soeria-Atmadja, Aikaterini Nasi, Maurizio Zazzi, Ilaria Vicenti, Lars Naver, Anna Nilsson, Francesca Chiodi

**Affiliations:** ^1^Department of Microbiology, Tumor and Cell Biology, Karolinska Institutet, Stockholm, Sweden; ^2^Department of Clinical Science, Intervention and Technology (CLINTEC), Karolinska Institutet, Stockholm, Sweden; ^3^Department of Pediatrics, Karolinska University Hospital, Stockholm, Sweden; ^4^Department of Medical Biotechnology, University of Siena, Siena, Italy; ^5^Department of Women’s and Children Health, Karolinska Institutet, Stockholm, Sweden

**Keywords:** vaccination, hepatitis B virus, hepatitis A virus, HIV-1 reservoirs, anti-retroviral therapy, immune activation, T-cell proliferation

## Abstract

During anti-retroviral therapy (ART) HIV-1 persists in cellular reservoirs, mostly represented by CD4+ memory T cells. Several approaches are currently being undertaken to develop a cure for HIV-1 infection through elimination (or reduction) of these reservoirs. Few studies have so far been conducted to assess the possibility of reducing the size of HIV-1 reservoirs through vaccination in virologically controlled HIV-1-infected children. We recently conducted a vaccination study with a combined hepatitis A virus (HAV) and hepatitis B virus (HBV) vaccine in 22 HIV-1-infected children. We assessed the size of the virus reservoir, measured as total HIV-1 DNA copies in blood cells, pre- and postvaccination. In addition, we investigated by immunostaining whether the frequencies of CD4+ and CD8+ T cells and parameters of immune activation and proliferation on these cells were modulated by vaccination. At 1 month from the last vaccination dose, we found that 20 out of 22 children mounted a serological response to HBV; a majority of children had antibodies against HAV at baseline. The number of HIV-1 DNA copies in blood at 1 month postvaccination was reduced in comparison to baseline although this reduction was not statistically significant. A significant reduction of HIV-1 DNA copies in blood following vaccination was found in 12 children. The frequencies of CD4+ (naïve, effector memory) and CD8+ (central memory) T-cell subpopulations changed following vaccinations and a reduction in the activation and proliferation pattern of these cells was also noticed. Multivariate linear regression analysis revealed that the frequency of CD8+ effector memory T cells prior to vaccination was strongly predictive of the reduction of HIV-1 DNA copies in blood following vaccination of the 22 HIV-1-infected children. The results of this study suggest a beneficial effect of vaccination to reduce the size of virus reservoir in HIV-1-infected children receiving ART. A reduced frequency of activated CD4+ cells and an increase in central memory CD8+ T cells were associated with this finding. Further studies should assess whether vaccination is a possible tool to reduce HIV-1 reservoirs.

## Introduction

Anti-retroviral therapy (ART) provided to HIV-1-infected patients infected with human immunodeficiency virus type 1 (HIV-1) does not lead to virus eradication as HIV-1 persists in memory CD4+ T cells which represent a stable and long-lived reservoir for this infection (1, 2). At present, approximately 40 million people live with HIV-1 infection and this number will increase as a result of new infections and ART scale-up limiting mortality. As HIV-1 patients need to receive life-long treatment, the economic burden of life-long ART for countries highly affected by HIV-1 is immense. The elimination of HIV-1 reservoirs could lead to cure of HIV-1 infection; accordingly, novel targets for curing HIV-1 infection through reactivation and elimination of virus reservoirs are under investigation. A possible approach is the “shock and kill” (3) strategy designed to induce the expression of HIV-1 proteins in infected host cells (“shock”) thereby enabling the immune system, primarily CD8+ cytotoxic T cells, to identify and clear these cells (“kill”). Histone deacetylases inhibitors (HDACis) were the first candidate compounds investigated as possible HIV-1 latency reversing agents. Clinical studies conducted with HDACis in HIV-1-infected patients have shown that HDACis may enhance HIV-1 transcription (4–8) although a significant decrease of virus reservoirs was not observed. A number of different compounds have later been considered, but the success in reducing the virus reservoir is still limited (9). Thus, additional approaches need to be developed and evaluated to reactivate and eliminate latent HIV-1 infection. Until new medicines will be available for curing HIV-1 infection it is however important to provide ART to HIV-1-infected patients as early as possible, ideally already at the phase of primary HIV-1 infection, as early ART administration reduces inflammation linked to HIV-1 pathogenesis and confines the size of HIV-1 reservoirs (10).

The possibility of developing an HIV-1 cure in the context of pediatric HIV-1 infection is also under debate (11). One of the obvious features of pediatric infection which may be advantageous in the context of HIV-1 cure is that HIV-1 infection can be diagnosed in infants born to HIV-1-infected mothers within hours from birth thus facilitating early ART intervention. However, when ART was discontinued in a group of HIV-1-infected children in South Africa who were provided ART from a median age of 7 weeks (12), only one child presented with undetectable virus in absence of drugs, whereas all other children presented with virus rebound (13). An additional recent publication reported HIV-1 virological remission in an HIV-1-infected teenager who started ART before 6 months of age (14); once this patient discontinued ART, at approximately 6 years of age, HIV RNA remained <50 copies/mL for approximately 12 years and CD4+ T-cell counts were stable (14).

Several phenotypic dysfunctions occur in the total bulk of T cells, HIV-1 specific and non-specific, during HIV-1 infection. T cells are characterized by the high expression of activation markers, including CD38 and HLA-DR, which are independent predictors of CD4+ T-cell decline and progression to AIDS (15). In HIV-1-infected children, the expression of activation markers was reduced after 44 weeks of ART (16). Divergent results have, however, also been presented on persistent CD8+ T-cell activation in HIV-1-infected youth after 48 weeks of ART (17). These findings suggest that strategies to eliminate HIV-1 reservoirs may also include strengthening CD8+ T-cell responses in HIV-1-infected individuals; a characteristic feature of HIV-1 pathogenesis is a dysfunctional pool of CD8+ T cells, considered to be one of the causes for establishment of chronic HIV-1 infection [reviewed in Ref. (18)].

The influence of vaccination in affecting the size of HIV-1 reservoirs has been previously evaluated in the context of influenza vaccination in cohorts of adult HIV-1-infected individuals (19–21). Whether vaccinations provided during childhood may be useful tools to reduce the size of HIV-1 reservoir in HIV-1-infected children has not been previously evaluated; this is important to study as childhood vaccinations are provided to the majority of infants worldwide. Vaccination against hepatitis B virus (HBV) has been introduced in many countries at birth; within the frame of a recent vaccination study against combined hepatitis A virus (HAV) and HBV infections in young HIV-1-infected children who had received ART for a prolonged period of time, we assessed whether the size of the virus reservoir and indicators of CD4+ and CD8+ T-cell function were modulated by HBV vaccination. In individuals naturally infected with HBV, the strength of CD8+ T-cell responses to the virus determines whether the infected individual clears the infection or HBV establishes a persistent infection (22, 23). In addition, a broad recognition range of HBV epitopes by CD4+ and CD8+ T cells is a prognostic marker for likelihood of resolving acute HBV infection (24, 25). Virological markers of HBV-related diseases have recently been associated with HLA-A genotype, further supporting the important role of CD8+ T cells in controlling natural HBV infection (26). MHC class-I-restricted HBV epitopes derived from several HBV proteins, including the HB antigen, have been shown to activate CD8+ T-cell responses (27).

## Materials and Methods

### Patient Cohorts and HBV Vaccination Schedule

Twenty-two HIV-1-infected children (8 males and 14 females) with a median age of 15 years (range 6–18 years) followed at Karolinska University Hospital were included in the vaccination study. For 20 of the children, a vertical route of transmission was confirmed; the route of transmission was unknown for two of the children. At the time of the first vaccination dose, the children had received ART for a median period of 7.2 years and their median CD4+ T-cell count was 715 cells/μL. Viral load was undetectable (<20 copies/mL) in plasma of 18 (81.8%) children.

HIV-1-infected children were vaccinated with Twinrix^®^ (GlaxoSmithKline AB), a combination vaccine against HAV (inactivated) and HBV (recombinant); the children received three doses of vaccine with 4-week intervals between the doses. According to clinical records, the children were not previously vaccinated for HBV. A blood sample was collected prior to vaccination and at 1 month after completed vaccination. Peripheral blood mononuclear cells (PBMCs) were isolated using Ficoll gradient centrifugation (Lymphoprep, Axis-Shield Poc AS, Oslo, Norway) and stored in liquid nitrogen at −160°C for later use. Serum specimens were stored at −80°C until further analyses were conducted.

The study was carried out in accordance with the recommendations of the ethical committee at the Karolinska Institutet (Protocol no. Dnr 2013/774-31/1). Written informed consent was obtained, in accordance with the Declaration of Helsinki, from the parents of study participants following a clear explanation of the study purpose, benefit and possible discomfort.

### Determination of Plasma Anti-HBV and Anti-HAV Antibodies

The anti-HBs Monolisa Anti-HBs Plus assay (Bio-Rad, France) was used to measure the plasma levels of anti-HBV vaccine antibodies. The assay was run according to the instruction provided by the manufacturer. The cutoff value for samples to be considered positive for the presence of antibodies to the HBV vaccine is 10 IU/L (1 log IU/L). All samples and standards were tested in duplicate. The OD values were converted to concentrations using Microplate manager version 6 (Bio-Rad, CA, USA).

Total (IgG and IgM) HAV antibodies, and IgM on a separate test, were measured by electro-chemiluminescence immunoassay (ECLIA) (Roche, Basel, Switzerland). Samples were considered positive for total anti-HAV Igs (IgG and IgM) antibodies when the measurements showed values >20 IU/L.

### Measurement of Total PBMC HIV-1 DNA

The PBMC DNA was obtained by manual extraction with the High Pure Viral Nucleic Acid Kit (Roche, Basel, Switzerland). Total PBMC HIV-1 DNA was quantified by using a homemade Taqman real-time assay targeting a highly conserved region of the long terminal repeat gene (28).

### Immunostainings of T-Cell Subpopulations

Peripheral blood mononuclear cells obtained pre- and postvaccination were thawed and stained simultaneously to avoid run-to-run variation. To measure the frequency of different T-cell populations and immune activation of T cells, the following monoclonal antibodies were used: anti-CD3 (UCHT1), anti-CD4 (RPA-T4), anti-CD8 (RPA-T8), anti-CD45RA (HI100), anti-CCR7 (3D12), anti-CD38 (HIT2), and anti-HLA-DR (G46-6) all from BD Biosciences (CA, USA). To assess T-cell proliferation, Ki67 expression was measured: for this purpose, PBMCs were fixed, permeabilized using the BD Cytofix/Cytoperm kit, and stained with anti-Ki67 (MIB-1) from Dako (Glostrup, Denmark). LIVE/DEAD Fixable Near-IR kit (Life Technologies Europe BV, Stockholm, Sweden) was used to exclude dead cells from the analyses. Stained cells were washed with phosphate-buffered saline (PBS) before being fixed in 2% paraformaldehyde. All antibodies were used at the concentrations determined after titration experiments. Matched isotype controls or fluorescence minus one (FMO) were used to set the gating strategies. Fluorescence intensities were measured with LSRII (BD) and data were analyzed using FlowJo, version 9.4.11 (Tree Star, OR, USA).

T-cell subsets were gated out using the following antibody combinations for CD4+ T-cell subsets: total CD4+ T cells (CD3+ CD4+ CD8−), CD4+ naïve (CD3+ CD4+ CD8− CD45RA+ CCR7+), CD4+ central memory (CM) (CD3+ CD4+ CD8− CD45RA− CCR7+), CD4+ effector memory (EM) (CD3+ CD4+ CD8− CD45RA− CCR7−), and CD4+ terminally differentiated effector memory (TEMRA) (CD3+ CD4+ CD8− CD45RA+ CCR7−). CD8+ T-cell subsets were defined as total CD8+ T cells (CD3+ CD4− CD8+), CD8+ naïve (CD3+ CD4− CD8+ CD45RA+ CCR7+), CD8+ CM (CD3+ CD4− CD8+ CD45RA− CCR7+), CD8+ EM (CD3+ CD4− CD8+ CD45RA− CCR7−), and CD8+ TEMRA cells (CD3+ CD4− CD8+ CD45RA+ CCR7−).

### Statistical Analysis

Data were analyzed using GraphPad prism version 7 (La Jolla, CA, USA). Paired *T*-test was used to analyze clinical and immunological parameters. A value of *p* < 0.05 was considered statistically significant.

Linear regression analysis was performed to identify clinical and immunological parameters as potential predictors of the change in HIV-1 DNA copies between baseline (BL) and 1 month postvaccination (M1). Due to the small sample size and the large number of variables showing association with the dependent variable at univariate analysis, only parameters scored as significant at *p* < 0.01 level at univariate analysis were selected for stepwise multivariate linear regression analysis. In the first step, CD4+ and CD8+ T cells and subsets were included. In the second step, the expression of activation markers (CD38 and HLA-DR) among CD4+ and CD8+ T cells and subsets were added; the expression of Ki67 proliferation marker among CD4+ and CD8+ T cells and subsets were entered in the third step. The number of HIV-1 DNA copies at BL were added in the fourth step. The values of *p* < 0.05 were considered statistically significant. Linear regression analysis was performed using SPSS version 25 (IBM Corp, Armonk, NY, USA).

## Results

### Antibody Response to HBV Vaccine and Size of the Virus Reservoir

**Figure 1:**
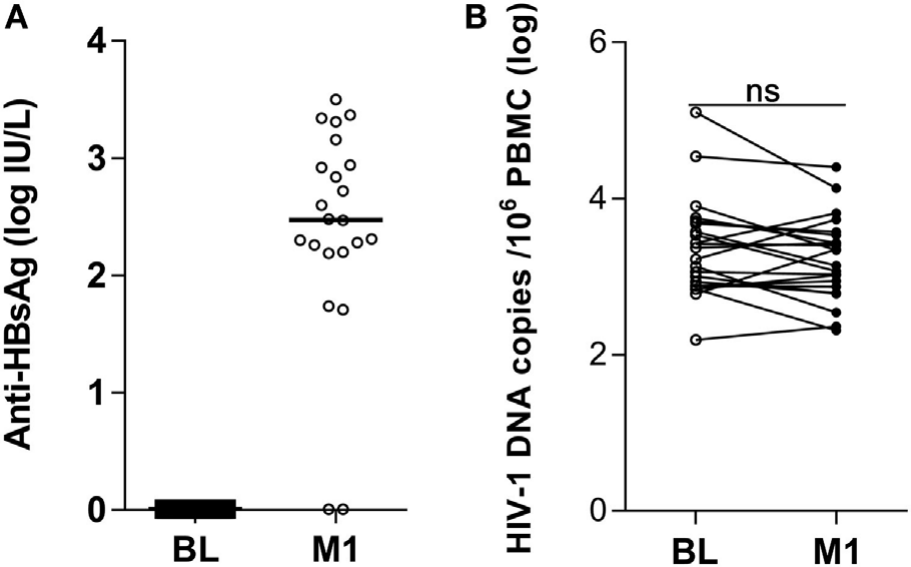
Antibody titers to hepatitis B virus vaccine and HIV-1 DNA copies in peripheral blood mononuclear cells (PBMCs) of HIV-1-infected children pre-vaccination and postvaccination. Antibodies to the HBsAg were detected in plasma **(A)** and HIV-1 DNA copies/10^6^ PBMCs were measured in PBMCs **(B)** from 22 HIV-1-infected children pre-vaccination and at 1 month postvaccination. ns, not significant; BL, baseline; M1, month 1.

Prior to vaccination, none of the 22 HIV-1-infected children included in the study displayed measurable levels of antibody to HBsAg (Figure 1A) and all samples scored below the cutoff value of 1 log IU/L. At 1 month from the last vaccination 20 of the 22 children had detectable antibody titers (>1 log IU/L) to HBsAg; the median logarithmic titer of anti-HBsAg antibody was 2.48 IU/L (range 0.01–3.50). The clinical characteristics of the individuals included in the study are shown in Table 1.

**Table 1:** Clinical characteristics of HIV-1-infected children included in the study.

Characteristic	HIV-1-infected baseline	HIV-1-infected M1
Age (years); median (range)	15 (6–18)	Idem
Gender		
Male	8 (36.4%)	
Female	14 (63.6%)	Idem
Transmission route		
Vertical	20 (90.9%)	
Other/unknown	2 (9.1%)	Idem
Years on ART: median (range)	7.2 (0.73–17.46)	Idem
CD4 count (cells/mL): median (range)	715 (250–1,360)	765 (240–1,390)
Viral load (RNA copies/mL)		
Undetectable	18 (81.8%)	20 (90.9%)
44, 155, 749, 96,500	4 (18.2%)	–
53, 511	–	2 (9.1%)
HIV DNA copies/10^6^ PBMC (log): mean (range)	3.35 (2.19–5.10)	3.23 (2.31–4.40)

*Undetectable viral load = <20 copies/mL*.

The analysis of total anti-HAV Igs (IgG and IgM) antibodies revealed that 12 of the 22 children included in the study had antibodies against HAV in serum prior to vaccination; all 22 children were positive for HAV total Ig following vaccination (data not shown). As the anti-HAV IgM test scored negative pre- and postvaccination, we concluded that 12 of the patients carried anti-HAV IgG at BL and all 22 at 1 month postvaccination. As it is not clear whether the anti-HAV antibodies detected at BL were mounted as results of previous exposure, this parameter was not further evaluated.

The size of the virus reservoir, expressed as HIV-1 DNA copies/10^6^ PBMCs, was measured prior to vaccination and at 1 month from the last vaccination (Figure 1B). The median logarithmic value (range) of HIV-1 DNA copies in blood cells of HIV-1-infected children was 3.29 (2.19–5.10) at BL and 3.10 (2.31–4.40) at 1 month postvaccination. The decline in number of HIV-1 DNA copies detected at 1 month postvaccination was not statistically significant. Likewise, no correlation could be found between the levels of anti-HBsAg antibodies at 1 month from last vaccination and the size of virus reservoir at either BL or 1 month postvaccination (results not shown).

### Frequency of Blood CD4+ and CD8+ T-Cell Subpopulations and Expression of Activation Markers in Vaccinated Individuals

**Figure 2:**
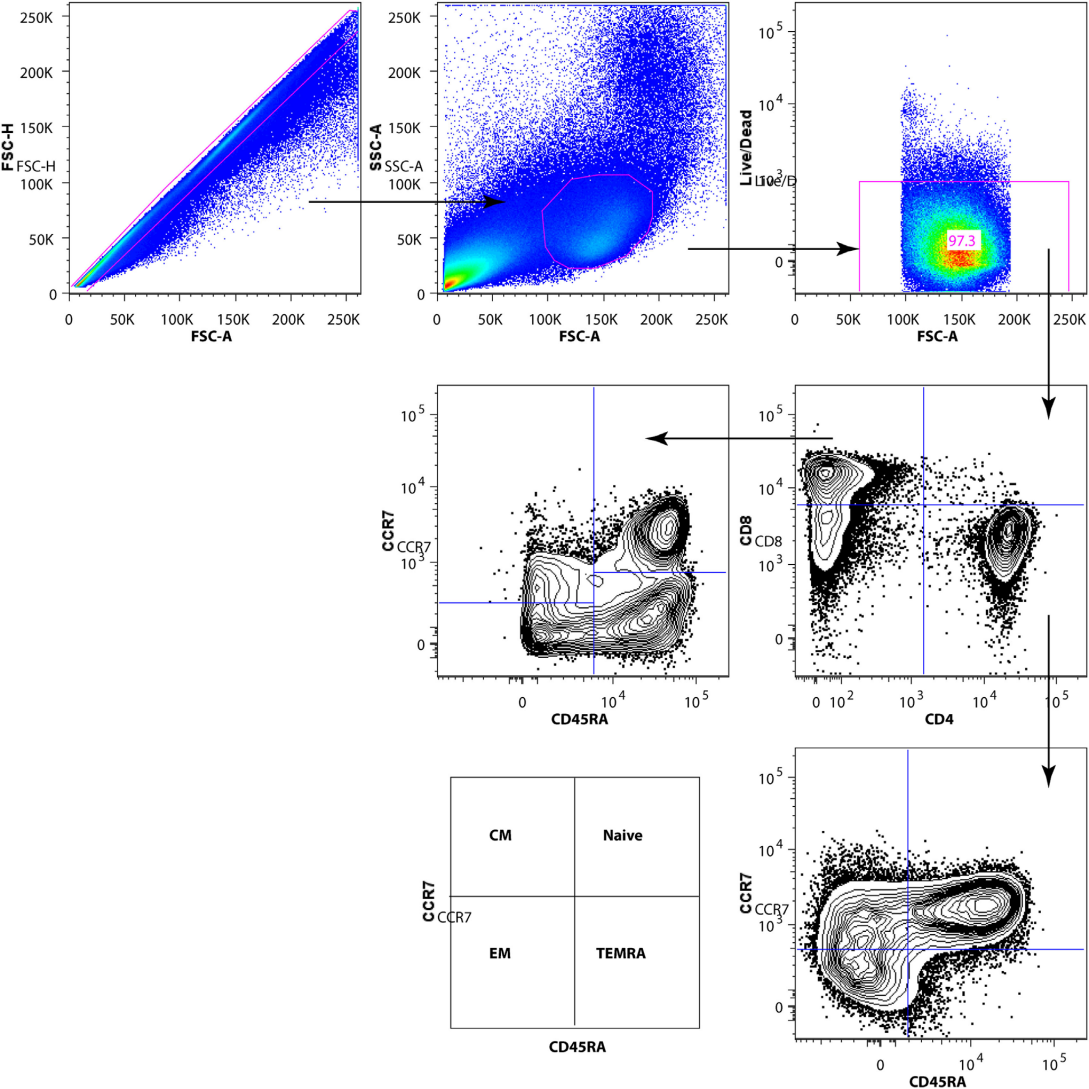
Gating strategy for subpopulations of CD4+ and CD8+ T cells. After exclusion of dead cells and doublets, total T cells were identified by CD3 surface expression. Gated CD3+ T cells were divided into CD4+ and CD8+ T-cell subsets. The CD4+ and CD8+ T-cell subpopulations [naive, EM, CM, TEMRA] were identified by CD45RA and CCR7 expression.

The gating strategy for characterization of CD4+ and CD8+ T-cell subpopulations is shown in Figure 2. We analyzed the frequency of CD4+ and CD8+ T-cell subpopulations in blood of HIV-1-infected children prior to vaccination and at 1 month from completed vaccination and also assessed the expression of activation markers CD38 and HLA-DR on CD4+ and CD8+ subpopulations.

**Figure 3:**
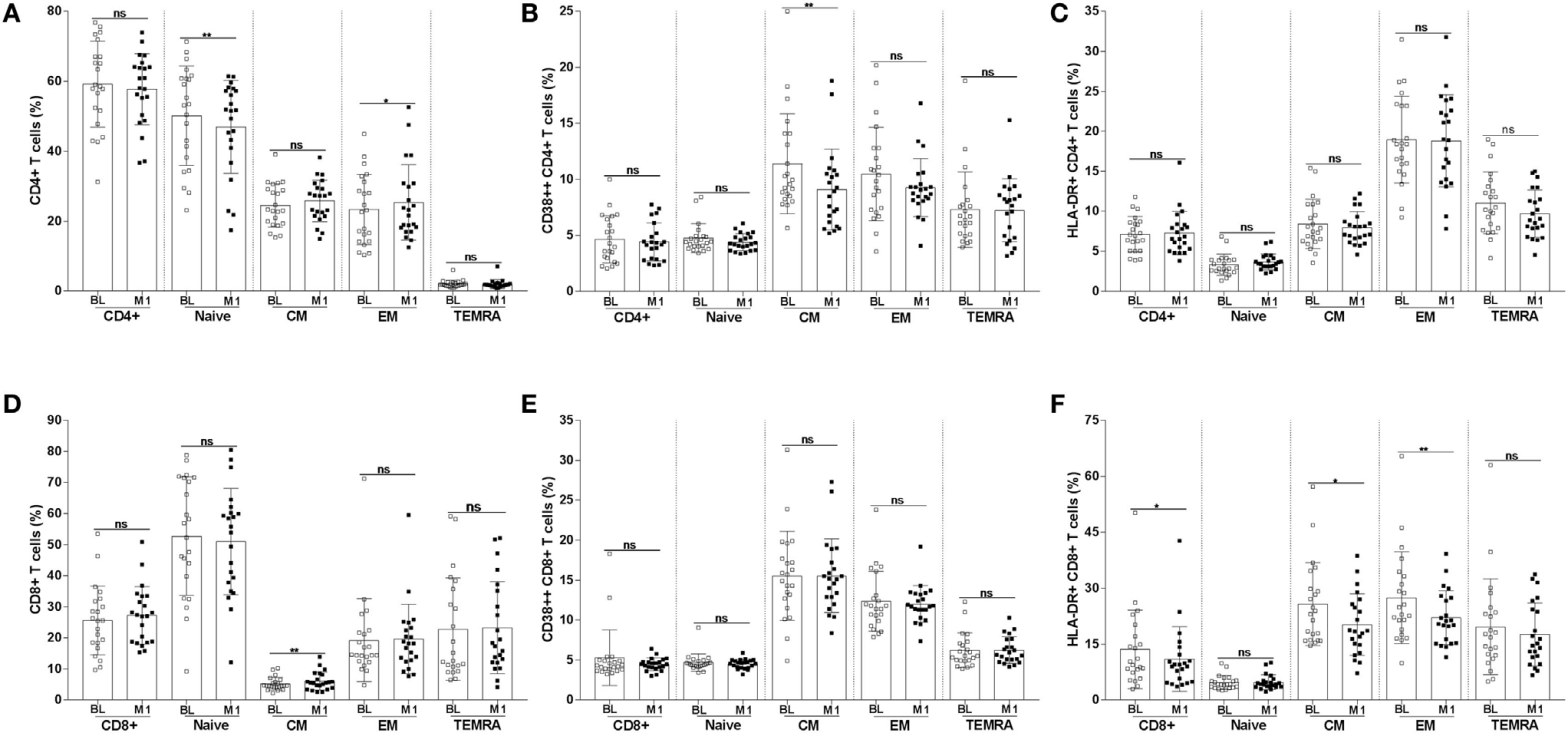
Frequency of CD4+ and CD8+ T-cell subpopulations and of T cells expressing the CD38 and HLA-DR activation markers. CD4+ T cells were divided into naïve, CM, EM, and TEMRA. The frequencies of CD4+ T-cell subpopulations **(A)** were assessed at BL and 1 month postvaccination. The frequency of CD4+ T-cell subpopulations expressing CD38 **(B)** and HLA-DR **(C)** were also measured. The frequency of CD8+ T-cell subpopulations and of CD8+ T cells expressing CD38 and HLA-DR are shown in panels **(D–F)**. *p < 0.05; **p < 0.01; ns, not significant; BL, baseline; M1, month 1.

The frequency of CD4+ T-cell subpopulations and the expression of activation markers on these cells are shown in Figures 3A–C. For some of the analyzed subpopulations, there was a significant difference between the samples collected at BL and at 1 month postvaccination. The frequency of naïve CD4+ T cells was reduced at month 1 from vaccination (mean value BL 50.2 versus M1 46.9; *p* < 0.01); the opposite trend was noticed for EM CD4+ T cells which frequency increased at 1 month from vaccination (mean value BL 23.3 versus M1 25.3; *p* < 0.05). The frequency of CM CD4+ T cells expressing the activation marker CD38 was significantly reduced at 1 month (mean value BL 11.4 versus M1 9.0; *p* < 0.01) (Figure 3B).

The frequency of CM CD8+ T cells (Figures 3D–F) significantly increased at month 1 (mean value BL 5.2 versus M1 6.1; *p* < 0.01). A decline in the frequency of cells expressing the activation marker HLA-DR was noticed among total (mean value BL 13.6 versus M1 11.0; *p* < 0.05), CM (mean value BL 25.7 versus M1 20; *p* < 0.05), and EM (mean value BL 27.4 versus M1 22.0; *p* < 0.01) CD8+ T cells.

**Figure 4:**
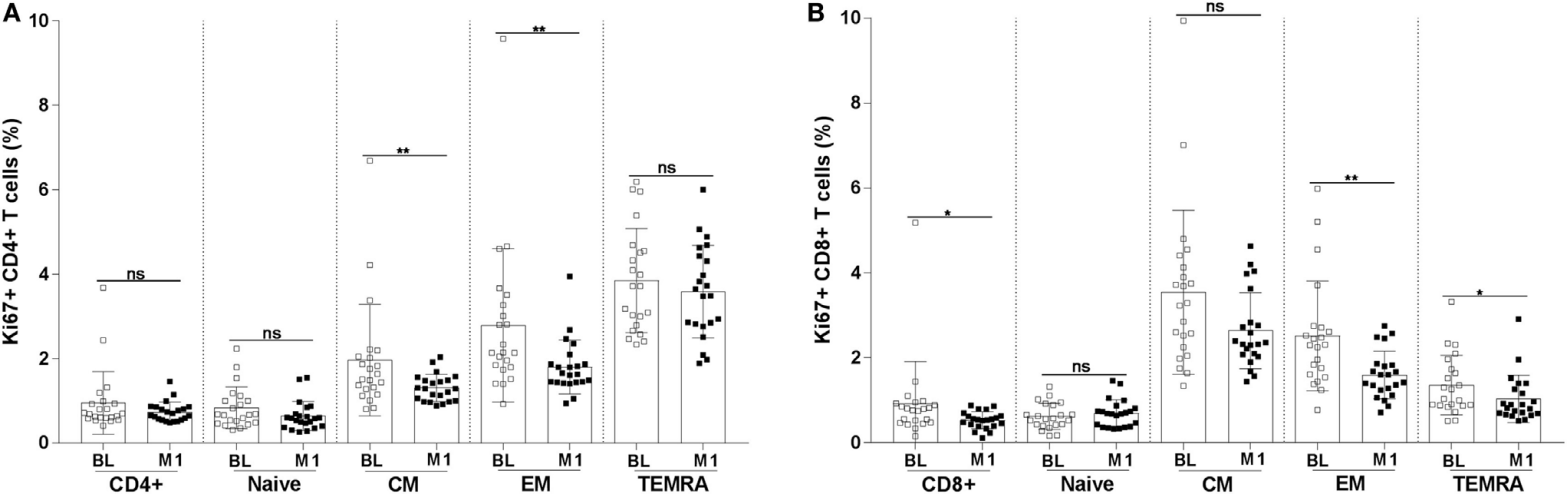
Frequency of Ki67+ CD4+ and CD8+ T-cell subpopulations. The frequency of Ki67+ CD4+ **(A)** and CD8+ **(B)** T-cell subpopulations (naïve, CM, EM, and TEMRA) were measured in blood specimens of 22 HIV-1-infected children at baseline and 1 month postvaccination. *p < 0.05; **p < 0.01; ns, not significant; BL, baseline; M1, month 1.

Abnormal proliferation of T cells is a sign of immunopathology during HIV-1 infection. In order to assess the degree of proliferation of CD4+ and CD8+ T-cell subpopulations, we measured the frequency of cells expressing the Ki67 proliferation marker. A decline in the frequency of cells expressing Ki67 was noticed for CM (mean value BL 1.96 versus M1 1.3; *p* < 0.01) and EM (mean value BL 2.8 versus M1 1.8; *p* < 0.01) CD4+ T cells (Figure 4A). A clear decline of cells expressing Ki67 was also noticed among CD8+ T cells at month 1 reaching a statistically significant difference for total (mean value BL 0.9 versus M1 0.5; *p* < 0.05), EM (mean value BL 2.5 versus M1 1.6; *p* < 0.01), and TEMRA (mean value BL 1.36 versus M1 1.0; *p* < 0.05) CD8+ T cells (Figure 4B).

### Factors Predictive of Changes in HIV-1 DNA Copies following Vaccination

In order to understand if changes in HIV-1 DNA copies postvaccination could be predicted by clinical and immunological parameters, we conducted linear regression analysis. At univariate analysis, age, CD4+ T-cell count, and length of ART did not significantly predict a change in HIV-1 DNA copies following vaccination. By contrast, BL HIV-1 DNA levels and a number of immunologic markers were significantly predictive of HIV-1 DNA changes (Table 2).

**Table 2:** Univariate linear regression analysis between changes in HIV-1 DNA copies [baseline minus 1 month from vaccination (dependent variable)] and immunological and clinical parameters (independent variables).

Model	Unstandardized coefficients	Standardized coefficients	*t*	*p*-Value

	*B*	Beta		
CD4+	−1,037.17	−0.53	−2.77	0.012
CD38+ CD4+	2,550.75	0.22	1.02	ns
HLA-DR+ CD4+	5,332.50	0.49	2.49	0.022
Naïve CD4+	−521.33	−0.31	−1.44	ns
CD38+ naïve CD4+	−941.69	−0.05	−0.23	ns
HLA-DR+ naïve CD4+	−2,283.45	−0.13	−0.56	ns
CM CD4+	−322.40	−0.08	−0.37	ns
CD38+ CM CD4+	941.17	0.17	0.79	ns
HLA-DR+ CM CD4+	3,691.52	0.47	2.39	0.027
EM CD4+	1,136.95	0.47	2.38	0.027
CD38+ EM CD4+	1,714.14	0.30	1.39	ns
HLA-DR+ EM CD4+	127.13	0.03	0.13	ns
TEMRA CD4+	3,111.49	0.14	0.62	ns
CD38+ TEMRA CD4+	−108.46	−0.02	−0.07	ns
HLA-DR+ TEMRA CD4+	852.69	0.14	0.62	ns
CD8+	1,247.43	0.57	3.10	0.006
CD38+ CD8+	5,686.74	0.82	6.38	0.000
HLA-DR+ CD8+	1,786.08	0.78	5.57	0.000
Naïve CD8+	−638.82	−0.50	−2.60	0.017
CD38+ naïve CD8+	1,785.66	0.08	0.36	ns
HLA-DR+ naïve CD8+	2,400.15	0.19	0.85	ns
CM CD8+	2,385.17	0.20	0.92	ns
CD38+ CM CD8+	2,828.93	0.65	3.84	0.001
HLA-DR+ CM CD8+	1,472.30	0.68	4.11	0.001
EM CD8+	1,565.03	0.87	7.75	0.000
CD38+ EM CD8+	4,258.01	0.66	3.92	0.001
HLA-DR+ EM CD8+	1,391.69	0.71	4.48	0.000
TEMRA CD8+	−214.19	−0.15	−0.66	ns
CD38+ TEMRA CD8+	5,343.73	0.48	2.42	0.025
HLA-DR+ TEMRA CD8+	654.77	0.35	1.66	ns
KI-67+ CD4+	14,341.00	0.44	2.20	0.040
KI-67+ naïve CD4+	20,125.01	0.41	2.01	ns
KI-67+ CM CD4+	6,922.45	0.38	1.83	ns
KI-67+ EM CD4+	3,022.22	0.23	1.04	ns
KI-67+ TEMRA CD4+	7,480.53	0.38	1.84	ns
KI-67+ CD8+	22,851.56	0.94	12.34	0.000
KI-67+ naïve CD8+	26,084.09	0.33	1.56	ns
KI-67+ CM CD8+	5,046.98	0.40	1.97	ns
KI-67+ EM CD8+	10,504.31	0.56	3.05	0.006
KI-67+ TEMRA CD8+	6,850.65	0.20	0.91	ns
Age	1,463.11	0.20	0.92	ns
DNA_BL	0.87	0.98	23.89	0.000
CD4_BL	−41.39	−0.43	−2.12	ns
Years ART	−377.89	−0.07	−0.31	ns

*ns, non-significant; significant values <0.01*.

When the significant variables were processed through stepwise multivariate linear regression analysis, five models were generated (Table 3) showing significant predictive value for the frequency of CD8+ EM T cells, CD38+ EM CD8+ T cells, HLA-DR+ CM CD8+ T cells, Ki67+ CD8+ T cells, and HIV-1 DNA copies prior to vaccination. In model 1, a high-regression coefficient was found for CD8+ EM T cells which explained 75% of the variance for the decline of HIV-1 DNA postvaccination. In the final model 5, the frequency of Ki67+ CD8+ T cells and HIV-1 DNA copies prior to vaccination retained the maximal predictive value; however, these variables only contributed with 5% of the variance for the decline of HIV-1 DNA postvaccination.

**Table 3:** Stepwise multivariate linear regression analysis between changes in HIV-1 DNA copies [baseline minus 1 month from vaccination (dependent variable)] and clinical and immunological parameters (independent variables).

Model		Unstandardized coefficients	Standardized coefficients	*t*	Sig.	*R*^2^	*R*^2^ change	Adjusted *R*^2^	*F* change (significance of *F* change)

		*B*	Beta						
1	EM CD8+	1,565.03	0.866	7.75	0.000	0.750	0.750	0.738	60.10***

2	EM CD8+	1,294.63	0.717	6.22	0.000	0.814	0.063	0.794	6.45*
	CD38+ EM CD8+	1,891.26	0.293	2.54	0.020				

3	EM CD8+	947.48	0.524	3.92	0.001	0.856	0.042	0.832	5.23*
	CD38+ EM CD8+	2,146.67	0.332	3.15	0.006				
	HLA-DR+ CM CD8+	586.97	0.270	2.29	0.034				

4	EM CD8+	396.01	0.219	1.87	0.079	0.932	0.076	0.916	19.14***
	CD38+ EM CD8+	974.18	0.151	1.77	0.095				
	HLA-DR+ CM CD8+	361.01	0.166	1.92	0.072				
	Ki67+ CD8+	13,719.01	0.564	4.38	0.000				

5	EM CD8+	179.14	0.099	1.51	0.152	0.981	0.049	0.976	42.58***
	CD38+ EM CD8+	209.73	0.032	0.66	0.520				
	HLA-DR+ CM CD8+	−76.80	−0.035	−0.63	0.537				
	Ki67+ CD8+	4,976.75	0.205	2.31	0.035				
	HIV-1 DNA copies	0.64	0.718	6.53	0.000				

*<0.05; ***<0.001.

**Figure 5:**
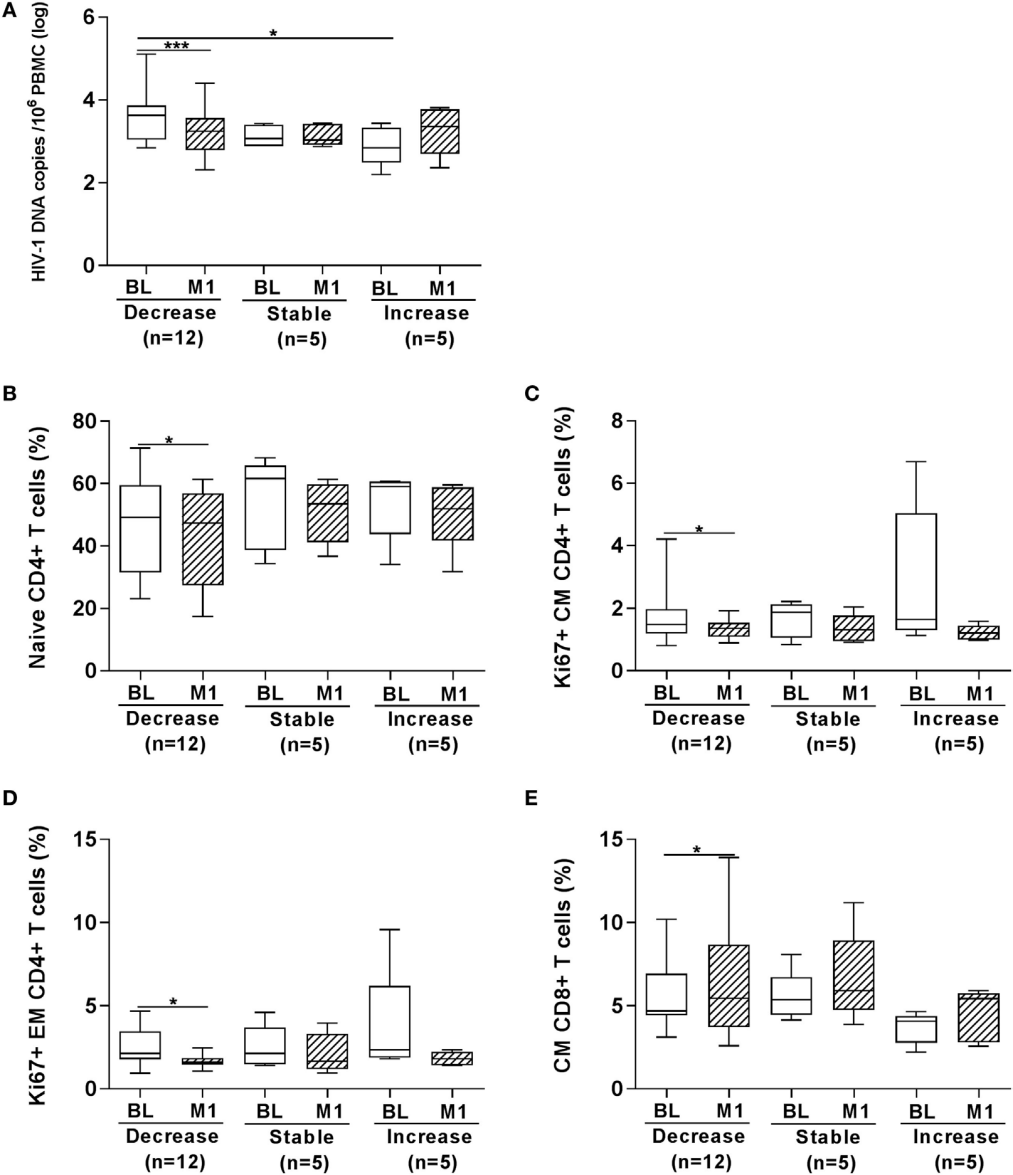
Frequency of T-cell subpopulations according to changes in HIV-1 DNA copies at 1 month from vaccination. HIV-1 DNA copies/10^6^ peripheral blood mononuclear cells (PBMCs) were measured in PBMCs from 22 HIV-1-infected children **(A)**. In the group “decrease,” 12 children are included who displayed >10% decrease in the number of HIV-1 DNA copies at 1 month from last vaccination as compared with BL value; in the group “stable,” 5 children who displayed minor variations (<10%) between the two time points are included; in the group “increase,” 5 children were included showing an increased (>10% variation) number of HIV-1 DNA copies at 1 month. In the three groups of children divided according to whether the HIV-1 DNA copies in PBMCs decreased, remained stable or increased at month 1 from vaccination we found that the frequency of naïve CD4+ T cells **(B)**, Ki67+ CM CD4+ T cells **(C)** and Ki67+ EM CD4+ T cells **(D)** was reduced in the “decrease” group; in the same group an increase in CM CD8+ T cells **(E)** was revealed at month 1. BL, baseline; M1, month 1. *p < 0.05; ****p < 0.001.

In order to further dissect whether HBV vaccination had an impact on the size of the virus reservoir, we also analyzed the number of HIV-1 DNA copies by separating patients in three groups (Figure 5) including the following: (i) children who displayed a significantly reduced (>10% variation) number of HIV-1 DNA copies at 1 month from last vaccination (median copies 3.25) as compared with BL logarithmic value (median 3.63, *p* < 0.001); (ii) children who displayed minor variation (<10%) between the two time points (month 1 copies 3.03 versus copies at BL 3.06); (iii) children who showed an increased (>10% variation) number of HIV-1 DNA copies at 1 month (median 3.35) as compared with BL (median 2.84). The three groups were on a median ART length of 7.5 (decrease), 6.4 (stable), and 7.3 (increase) years. As shown in Figure 5A, following vaccination a decreased level of HIV-1 DNA copies was found in 12 children; the level of HIV-1 DNA copies remained stable (or unchanged) in five children and increased in five children. The number of HIV-1 DNA copies was significantly higher prior to vaccination in the group “decrease” as compared with the group “increase.”

We plotted the frequency of T-cell subpopulations and expression of activation (CD38 and HLA-DR) and proliferation (Ki67) markers according to whether the children displayed a decrease, an increase, or a similar value of HIV-1 DNA copies in PBMCs at 1 month postvaccination compared with BL. Significant differences were found for four parameters (Figures 5B–E). The frequency of naïve CD4+ T cells was significantly lower in the patients displaying a decrease of DNA copies at 1 month postvaccination and a decline in Ki67+ expressing cells was also observed among CM and EM CD4+ T cells in this group of patients (Figures 5B–D). On the other hand, the frequency of CM CD8+ T cells was higher at 1 month postvaccination in the children displaying a decrease of DNA copies at 1 month postvaccination (Figure 5E).

## Discussion

Following vaccination with a mixed HAV and HBV vaccine in 22 HIV-1-infected children, we noticed a decline of total HIV-1 DNA in PBMCs; this decline, detected at 1 month postvaccination, was not statistically significant. In 54% of the HIV-1-infected children, however, a reduction in the number of HIV-1 DNA copies in PBMCs following vaccination was observed. The decline of HIV-1 DNA copies was detected in children presenting with a reduced frequency of proliferating CM CD4+ T cells and an increased frequency of CM CD8+ T cells following vaccination; this result may suggest that the declined levels of HIV-1 DNA copies in blood of HIV-1-infected children vaccinated with HAV and HBV may be due to a reduction of HIV-1 target cells in the presence of a larger number of CD8+ T cells flushing HIV-1-infected cells.

Previous studies have analyzed the possibility of affecting the size of the virus reservoir through influenza vaccination in cohorts of adult HIV-1-infected individuals. In a study conducted prior to ART introduction, major changes in the levels of pro-viral DNA in PBMCs could not be detected following vaccination; the levels of peak PBMC viral RNA, however, increased significantly following vaccination (19). A study conducted in 19 HIV-1-infected patients on ART showed that a transient elevation of plasma HIV-1 RNA levels occurred in patients with undetectable HIV-1 RNA following vaccination; an even more significant HIV-1 RNA increase was detected in patients presenting with detectable HIV-1 RNA at BL (20). Interestingly, in the latter study, the levels of HIV-1 DNA decreased in patients presenting with <400 HIV-1 RNA copies at BL (20); this result may indicate that, in the presence of effective ART, vaccination may reduce HIV-1-associated immune activation and lead to reduced HIV-1 replication. A very recent study conducted in HIV-1-infected women reported no changes in HIV-1 DNA concentration following influenza vaccination and, in addition, analysis of HIV-1 sequences did not suggest HIV-1 replication taking place as a result of vaccination (21).

Several measurements, including the copies of integrated HIV-1 DNA, can be used to evaluate the size of the virus reservoir (29); the measure of total HIV-1 DNA in PBMCs used in the present paper has been shown to correlate well with the inducible HIV-1 reservoir (30). Which of the components included in the vaccine can be correlated to the beneficial effect on the virus reservoir was not studied. The vaccine used in this study is a combination of inactivated HAV and recombinant HBV antigens and contains also a proportion of aluminum adjuvants provided as aluminum phosphate and hydroxide. It is obviously important to identify which vaccine components may have an impact on the size of the virus reservoir and future clinical studies should be designed to address this question. It is not easy to envisage why the vaccine used in this study should work to reduce the size of the virus reservoir in some patients when vaccines to other antigens, including influenza, did not induce any reduction in the size of virus reservoir. It cannot be excluded that the reduction of HIV-1 DNA upon vaccination noticed in 54% of the children may be due to reasons other than vaccination, e.g., ART treatment; further studies on this topic should therefore include a group of non-vaccinated, HIV-1-infected children, with similar length of treatment as the vaccinated group. Recent publications (31, 32) have provided a detailed picture of HIV-1 DNA decay dynamics in blood over time in both adults and children infected with HIV-1. The study in adults (31) showed that HIV-1 DNA decay comprises three different phases: phase I, from 0 to 1 year of ART, with a significant decay (approximately 1 log); phase II, from 1 to 4 years of ART, with <0.5 log decay; and phase III, after 4 years of ART, where the decay is minimal. In the study addressing HIV-1 DNA decay in the blood of HIV-1-infected children (32), patients were followed up to 4 years from ART initiation: a highly significant decay (1 log) was noticed during the first year of ART and the difference in HIV-1 DNA copies between 1 and 4 years of ART started to reach a plateau (0.2 log difference). In our cohort, at the time of vaccination, the children had been on ART for already a median time of approximately 7 years; this makes it possible that the reduction of HIV-1 DNA copies in blood noticed in some of the HIV-1-infected children may be due to effects promoted from vaccination An additional study, addressing long- and short-term dynamics of HIV-1 reservoir in peripheral blood, also reported that the copies of HIV-1 DNA remained stable over time (30).

The efficacy of HBV vaccines depends on the establishment of long-term immunological memory; the development of antibodies to protein-based HBV vaccine is the important hallmark for protection although HBV-DNA-based vaccines have been shown to stimulate CD8+ CTL cells (33, 34). It is likely that a well-controlled clinical picture of HIV-1 infection and sustained virological remission play an important role in decreasing the size of HIV-1 reservoir in vaccinated children. It is important to notice that previous studies addressing the potential role of vaccination in affecting the size of HIV-1 reservoirs were conducted in adults and this is, to the best of our knowledge, the first attempt to study whether vaccination has a role in modifying the dynamics of HIV-1 DNA in PBMCs of infected children.

Abnormal immune activation is a driving force for HIV-1 pathogenesis; prior to the introduction of ART in HIV-1 clinical practice, both CD4+ and CD8+ T cells were characterized by the high expression of surface activation markers, including CD38 and HLA-DR (35). ART introduction during the chronic phase of HIV-1 infection has reduced, but not normalized, abnormal features of immune activation and this ameliorated picture has also been detected in HIV-1-infected children treated with ART (36). In order to preserve immune competence and confine inflammation related to chronic HIV-1 infection, WHO recently recommended that ART should be initiated in HIV-1-infected children from birth and in adults as soon as infection is detected, ideally already during the phase of primary HIV-1 infection. To which extent ART initiation in the early phase of HIV-1 infection will prevent the establishment of abnormal immune activation is still an intensive topic of investigation and a recent study suggested that low levels of immune activation and inflammation may persist even when ART is initiated during the acute phase of HIV-1 infection (37). We recently reported that an equivalent abnormal expression of activation (HLA-DR and CD38 on CD4+ T cells) and terminal differentiation (CD127 on CD8+ T cells) markers were present on T cells from HIV-1-infected patients who initiated ART either during the primary or chronic phase of infection (38); the size of total HIV-1 DNA copies in blood of patients who initiated ART during primary infection was, however, lower compared with patients who initiated ART during chronic infection. Only a minority of the children included in our cohort had been treated from birth.

We noticed a significant fluctuation in terms of T-cell populations following vaccination: naïve CD4+ T cells were significantly reduced whereas the frequency of CM CD8+ T cells was significantly increased. The frequency of cells expressing parameters of immune activation and proliferation was reduced postvaccination with a decline in the frequency of activated CD38+ CM CD4+ T cells, HLA-DR+ CM and EM CD8+ T cells, Ki67+ CM and EM CD4+ T cells, and Ki67+ EM and TEMRA CD8+ T cells. CM CD4+ T cells are considered to be one of the major size HIV-1 reservoirs (39); it is noteworthy that vaccination led to a reduction in the frequency of CD38+ CM CD4+ T cells and that proliferating Ki67+ cells were reduced among both CM and EM CD4+ T cells. All in all, a reduction in the T-cell activation profile was found following vaccination in these virologically controlled HIV-1-infected children.

We also analyzed whether significant changes in the frequency of T-cell subpopulations and cells expressing activation and proliferation markers could be noticed in relation to the decrease of HIV-1 DNA copies in PBMCs following vaccination. It is of interest that in the group of children where a significant reduction of HIV-1 DNA copies was found following vaccination, there was an increase in CM CD8+ T cells and a reduction in the expression of Ki67 in CM and EM CD4+ T cells. According to this result it is likely that ameliorated immunological conditions, including the increased number of memory CD8+ T cells, cells devoted to the control of infections, may lead to a reduced amount of HIV-1 DNA copies in PBMCs. This result may reflect direct killing of infected cells due to increased frequency of CD8+ T cells or a dependency of the virus reservoir persistence on parameters of immune activation and proliferation. In this respect it is interesting that Hurst (40) showed that the expression of HLA-DR and CD38 on CD4+ and CD8+ T cells correlated with the size of total HIV-1 DNA at primary HIV-1 infection. Although it is not completely understood how HIV-1 reservoirs are maintained in HIV-1-infected patients during ART, a recent study suggested that these reservoirs are in part sustained by homeostatic cell proliferation (41). Thus, it appears relevant that the reduction of virus reservoir upon vaccination in our study occurs in parallel with the frequency of activated, proliferating CM CD4+ T cells.

In the group of 22 children in our study, the frequency of CD8+ EM T cells was an efficient predictor of the decline in HIV-1 DNA copies postvaccination using multivariate linear regression analysis. A study conducted in rhesus monkeys infected with simian immunodeficiency virus (SIV) showed that a strong viral inhibition activity was associated with EM CD8+ T cells rather than CM CD8+ T cells, high degranulation activity and perforin production by EM CD8+ T cells (42). Experiments conducted with sorted CD8+ T cells from elite controllers cocultured with HIV-1-infected CD4+ T cells revealed that EM CD8+ T cells had a high viral inhibition capacity in culture (43).

The results obtained in this study are important to elucidate which immunological markers can define ART-treated children where the HIV-1 reservoir can be reduced following a combined HAV and HBV vaccination. A follow-up by additional vaccination studies conducted in HIV-1-infected adults and children is important taking in consideration the time of ART initiation and other parameters currently utilized to evaluate the size of the virus reservoir (29).

## Ethics Statement

The study was carried out in accordance with the recommendations of the ethical committee at the Karolinska Institutet (Protocol no. Dnr 2013/774-31/1). Written informed consent was obtained, in accordance with the Declaration of Helsinki, from the parents of study participants following a clear explanation of the study purpose, benefit and possible discomfort.

## Author Contributions

YB acquired, analyzed, and interpreted the data for the work; drafted the work; and revised it for important intellectual content. RG acquired, analyzed, and interpreted the data for the work; and drafted the work. SS-A, MZ, LN, and Anna N gave substantial contributions to the conception of the work and revised the work for important intellectual content. Aikaterini N acquired, analyzed, and interpreted the data for the work; and revised the work for important intellectual content. IV acquired, analyzed, and interpreted the data for the work; and revised the work for important intellectual content. FC gave substantial contributions to the conception and design of the work and to the interpretation of data; and drafted the work and revised it for important intellectual content. All authors gave their final approval of the version to be published and agree to be accountable for all aspects of the work in ensuring that questions related to the accuracy or integrity of any part of the work are appropriately investigated and resolved.

## Conflict of Interest Statement

The authors declare that the research was conducted in the absence of any commercial or financial relationships that could be construed as a potential conflict of interest. The reviewer DT and handling Editor declared their shared affiliation.
